# Effects of surface treatments, LTD aging, and pH on the mechanical behavior of high-translucent zirconia

**DOI:** 10.1186/s12903-025-07473-2

**Published:** 2025-12-10

**Authors:** Secil Ozkan Ata, Sevgi Cetintas

**Affiliations:** Department of Prosthodontics, Faculty of Dentistry, Osmangazi University, Eskisehir, Turkey

**Keywords:** High-tranclucent zirconia, Esthetic dentistry, Colour stability

## Abstract

**Backgrounds:**

This study aims to evaluate the effects of low-temperature degradation (LTD) aging and pH variations on the mechanical and surface properties of 4Y-TZP and 6Y-TZP high-translucent zirconia materials subjected to different surface treatments.

**Methods:**

A total of 336 Y-TZP specimens (1.2 mm thickness, 12 mm diameter) were prepared and divided into two zirconia groups (4Y-TZP, 6Y-TZP) and four different surface treatment subgroups (glazed, grinding, grinding + polishing, grinding + glazing) (*n* = 42 per subgroup). Control specimens were stored in distilled water for 28 days (*n* = 14 per subgroup), while the remaining samples underwent LTD aging in an autoclave for 30 h. After aging, the specimens were immersed in pH 3 (acidic) and pH 9 (basic) solutions for 14 days. The mechanical and surface properties were assessed using biaxial flexural strength testing, Weibull analysis, X-ray diffraction phase analysis, scanning electron microscopy, and digital profilometry for surface roughness measurements.

**Results:**

The findings demonstrated that LTD aging combined with pH 3 exposure significantly affected the mechanical properties and phase transformation of Y-TZP zirconia (*p* < 0.05). Among the different surface treatments, grinding followed by polishing resulted in the highest biaxial flexural strength and lowest surface roughness, whereas grinding alone led to a significant decrease in mechanical performance. Grinding followed by glazing provided partial protection against surface degradation; however, the combination of LTD aging and pH exposure resulted in an overall reduction in mechanical strength across all groups. The 6Y-TZP group, due to its higher cubic phase content, exhibited greater resistance to LTD but showed lower biaxial flexural strength compared to 4Y-TZP. Additionally, pH 3 conditions induced more severe surface degradation and mechanical weakening, whereas pH 9 conditions resulted in more controlled degradation.

**Conclusion:**

Surface treatments, LTD aging, and pH variations play a critical role in determining the mechanical stability and surface characteristics of Y-TZP zirconia. Grinding followed by polishing is recommended for enhancing restoration durability in clinical applications. Acidic environments (pH 3) can negatively impact mechanical properties, while basic conditions (pH 9) provide comparatively better stability. The optimization of surface treatments and protective clinical strategies is essential to mitigating the adverse effects of LTD and ensuring the long-term success of zirconia restorations.

**Supplementary Information:**

The online version contains supplementary material available at 10.1186/s12903-025-07473-2.

## Introduction

The mechanical and optical characteristics of yttria-stabilized tetragonal zirconia polycrystals (Y-TZP) vary based on their yttria (Y₂O₃) concentration. Traditionally, 3Y-TZP has been extensively utilized in clinical applications because to its superior mechanical strength. As aesthetic demands have escalated, new transparent zirconia materials, including 4Y-TZP, 5Y-TZP, and 6Y-TZP, have been developed [1–3]. Despite 6Y-TZP’s superior translucency, its diminished mechanical strength constrains its clinical uses [4, 5]. The reaction of these advanced zirconia materials to low-temperature deterioration (LTD) aging and their enduring mechanical stability is not fully comprehended.

The LTD process may result in a progressive decline in the mechanical strength of Y-TZP materials over time. This transformation involves the transition from the tetragonal phase to the monoclinic phase, resulting in microcrack development, mechanical degradation, and heightened surface roughness [3, 5]. LTD can adversely impact the long-term durability of zirconia, thereby diminishing clinical success rates [6–8]. The long-term clinical performance of zirconia restorations is affected by chemical variables in the oral environment, in addition to mechanical aging [9, 10]. The pH levels in the oral cavity vary owing to dietary practices, stomach acid reflux, and oral hygiene products. Extended exposure to acidic (pH 3–4) or basic (pH 9–10) solutions can result in microstructural changes and a decline in mechanical characteristics [11, 12]. Acidic drinks, fruit juices containing citric acid, and exposure to stomach acid can lead to the breakdown of zirconia, facilitating phase change, surface wear, and fracture development. Alkaline conditions, such as some mouthwashes and cleaning chemicals, may influence the hydrothermal stability of zirconia and elevate surface roughness [12–14]. Nevertheless, research assessing the prolonged impact of varying pH levels post-LTD is few. Comprehending the influence of differing pH levels on the mechanical and surface characteristics of zirconia restorations is essential for evaluating their clinical durability.

Surface treatments are essential for altering the mechanical characteristics, surface quality, and adhesive bonding capability of zirconia restorations [15, 16]. Conventional surface treatments, including grinding, post-grinding polishing, and post-grinding glazing, affect the mechanical stability and aging resistance of zirconia. Nonetheless, there is inadequate scientific information about which surface treatment yields optimal performance following LTD exposure. Consequently, thorough investigations are necessary to ascertain the impact of various surface treatments on mechanical strength and surface roughness after LTD aging [16–18].

Although several studies have examined the effects of LTD on mechanical characteristics, the variations among different zirconia compositions and the influence of surface treatments on post-LTD stability are yet inadequately addressed [17–19]. In the present study, 4Y-TZP and 6Y-TZP were selected as representative categories of high-translucent zirconia, reflecting the strength–translucency trade-off. While 4Y-TZP provides higher strength with moderate translucency, 6Y-TZP offers enhanced translucency at the expense of strength. 5Y-TZP was deliberately excluded to enable a direct comparison between the two extremes of the translucency–strength spectrum. Although direct optical measurements were beyond the scope of this study, the designation ‘high-translucent’ follows the manufacturers’ classification of these materials. This work seeks to assess the alterations in mechanical strength and surface properties of 4Y-TZP and 6Y-TZP materials after LTD aging. Specimens will be submerged in pH 3 and pH 9 solutions for 14 days, and their biaxial flexural strength, phase composition (XRD), and surface morphology will be evaluated. The study hypothesis suggests that various surface treatments will significantly influence mechanical performance. Specifically, post-grinding polishing is expected to improve mechanical strength and diminish surface roughness, hence enhancing the longevity of zirconia restorations.

## Materials and methods

### Sample preparation

In this study, two types of high-translucent zirconia materials, 4Y-TZP and 6Y-TZP, were used. A total of 336 disc-shaped specimens were prepared, with 168 specimens per zirconia group (4Y-TZP: Nacera DD cube ML HT, Germany; 6Y-TZP: Nacera Pearl Q3 ML-HT, Germany). Sample size was determined and calculated using statistical power analysis (G*Power, version 3.1, Heinrich Heine University, Düsseldorf, Germany). A medium effect size (Cohen’s d = 0.5), a power of 80% (β = 0.20), and a significance level of α = 0.05 were considered in sample size determination.

The pre-sintered zirconia ceramics were sliced into plates measuring approximately 14.6 × 14.6 × 3.5 mm (ISO et1000, Buehler Ltd., USA). The dimensions of the cut specimens were selected considering the approximately 20% shrinkage that occurs during high-temperature sintering. The final specimen dimensions were 12 mm in diameter and 1.2 mm in thickness (disc-shaped), suitable for microstructural assessment and biaxial flexural strength testing using the piston-on-three-balls method. Specimens were sintered to full density at 1450 °C with a holding time of 120 min and a heating/cooling rate of 10 °C/min. This protocol corresponds to the conventional sintering program recommended by the manufacturers of both zirconia materials and is also supported by previous literature as an effective method to achieve optimal mechanical properties and microstructural stability [2, 3].

The final specimen dimensions (12 mm in diameter, 1.2 mm in thickness) were verified using a high-precision 150-mm digital caliper, considering the expected 20% shrinkage after sintering (Table 1).

**Table 1:** Summary of the experimental groups according to surface treatments and aging conditions

		glazed	grinded	Grinded + polished	Grinded + glazed
4YTZP	Control	4GC	4GrC	4GPC	4GGC
	LTD + pH3	4G3	4Gr3	4GP3	4GG3
	LTD + pH9	4G9	4Gr9	4GP9	4GG9
6YTZP	Control	6GC	6GrC	6GPC	6GGC
	LTD + pH3	6G3	6Gr3	6GP3	6GG3
	LTD + pH9	6G9	6Gr9	6GP9	6GG9

*Abbreviations*: *4G / 6G* Glazed 4Y-TZP / 6Y-TZP, *Gr* Grinded, *GP* Grinded + Polished, *GG* Grinded + Glazed, 3 = Low-temperature degradation (LTD) in pH 3, 9 = LTD in pH 9

### Surface treatments

To simulate various clinical applications, four different surface treatments were applied to the zirconia specimens: glazed (control group), grinding, grinding followed by polishing, and grinding followed by glazing (Table 2).

**Table 2:**
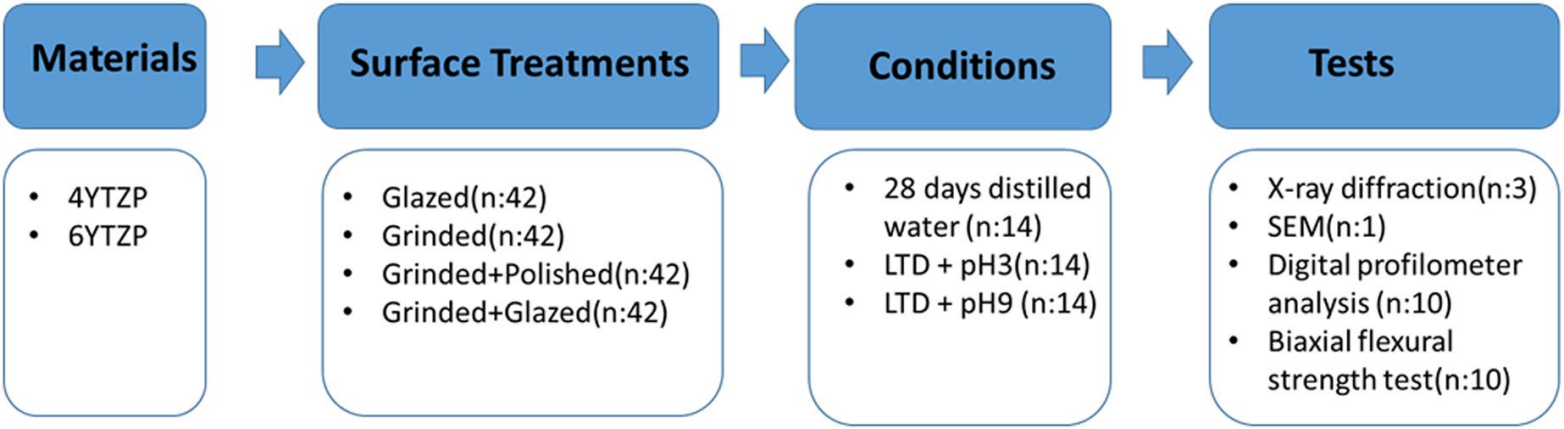
Experimental design table

### Glazed

Hydrothermal aging was performed by storing the specimens in an autoclave (134 °C, 2 bar) for 20 h, followed by immersion in acidic (pH 3) or alkaline (pH 9) buffer solutions for 14 days. For comparison, a control group was formed using glazed specimens that were not subjected to either hydrothermal aging or pH immersion. Ten specimens from each zirconia type (4Y-TZP and 6Y-TZP) served as controls.

The specimens were subjected to a standard glaze application according to the manufacturer’s instructions. IPS e.max Ceram Glaze (Ivoclar Vivadent, Liechtenstein) was applied as a thin layer using a brush and then fired in a Programat P510 furnace (Ivoclar Vivadent, Liechtenstein) at 800 °C for 1 min. The uniformity of the glaze layer was verified under a stereomicroscope (×20 magnification) to confirm homogeneous coverage across the specimen surface. All specimens were glazed in a single cycle under identical furnace conditions to ensure reproducibility. The glazing was performed using one firing cycle in a dental porcelain furnace (Programat P310, Ivoclar Vivadent, Liechtenstein) at 800 °C, with a heating rate of 55 °C/min and a holding time of 1 min. This single-cycle glazing protocol was selected in accordance with the manufacturer’s recommendation [20]. Standard heating and cooling cycles were applied to prevent internal stress formation. After completion, specimens were ultrasonically cleaned in distilled water using a Bandelin Sonorex (Germany) ultrasonic cleaner and air-dried (*n* = 42 per zirconia group).

### Grinding

The grinding process was performed using a high-speed dental handpiece (KaVo Extra Torque 605; KaVo do Brazil Ind. Com. Ltd) operating at 350,000 rpm. A fine-grit diamond bur (46 μm, #3101F; KG Sorensen, Brazil) was used for grinding.

To ensure standardization, specimens were fixed in a customized stabilization device and positioned parallel to the horizontal plane. A controlled force of 2 *N* ± 0.5 N was applied to ensure uniform grinding for each specimen. A new diamond bur was used for each specimen, and the grinding duration was limited to 30 s. The removal depth was set to 0.3 mm, and the final thickness was verified using a digital caliper (500-144B; Mitutoyo, Japan) with ± 0.01 mm precision. To prevent overheating and unintended phase transformation, water cooling (30 mL/min) was applied during the grinding process.

After grinding, specimens were ultrasonically cleaned in 99% isopropyl alcohol for 3 min and then air-dried (*n* = 42 per zirconia group).

### Grinding + polishing

The polishing process was performed after grinding to improve surface quality and reduce surface roughness. ZR-Diamond finishing burs (Komet, Germany) were used with a T2 REVO R170 contra-angle handpiece (Sirona, Bensheim, Germany) and a low-speed handpiece (KaVo Dental, Biberach, Germany) at 170,000 rpm under water cooling (30 mL/min). Medium-grit diamond burs (46 μm) were used for initial surface finishing.

To remove surface irregularities caused by grinding and enhance the polishing effect, a controlled additional grinding of 0.1 mm was performed, verified using a digital caliper. The polishing process consisted of a three-step protocol:

1. Coarse polishing: Meisinger ZR Polisher (Meisinger, Germany) at 10,000 rpm
2. Intermediate polishing: Optrafine System (Ivoclar Vivadent, Liechtenstein) at 12,000 rpm
3. Fine polishing: Signum HP Diamond (Kulzer, Germany) with a diamond suspension paste at 15,000 rpm

Polishing burs were sequentially used with Z-Shine (Dental Creations Ltd., USA) polishing discs, applying controlled pressure and time parameters. After the polishing process, specimens were ultrasonically cleaned in 99% isopropyl alcohol for 3 min and air-dried (*n* = 42 per zirconia group).

#### Grinding followed by glazing

The grinding process was performed using ZR-Diamond finishing burs (Komet, Germany) with a T2 REVO R170 contra-angle handpiece (Sirona, Bensheim, Germany) and a low-speed handpiece (KaVo Dental, Biberach, Germany). The procedure was conducted at 170,000 rpm, under water cooling at 30 mL/min, using medium-grit diamond burs (46 μm) to ensure uniform surface modification.

Following the grinding process, IPS e.max Ceram Glaze (Ivoclar Vivadent, Liechtenstein) was applied as a thin layer using a brush. The specimens were then sintered in a Programat P510 furnace (Ivoclar Vivadent, Liechtenstein) at 800 °C for 1 min. Standard heating and cooling cycles were implemented to prevent internal stress formation. After the glazing procedure, specimens were ultrasonically cleaned in distilled water using a Bandelin Sonorex (Germany) ultrasonic cleaner and air-dried (*n* = 42 per zirconia group). Following grinding and the other surface treatments (glazed, grinded + polished, grinded + glazed), XRD analysis was performed to evaluate possible tetragonal-to-monoclinic phase transformations, as illustrated in Fig. 3.

## Hydrothermal aging (LTD) and pH immersion protocol

Ten specimens from each surface treatment group were designated as the control group and stored in distilled water at 37 °C for 30 days. This group served as a reference for comparison with specimens subjected to LTD aging and pH exposure. The in vitro LTD aging was performed in an autoclave (STR-130, Tek-Bal, Türkiye) at 134 °C and 0.2 MPa (2 bar) pressure for 30 h. This accelerated aging method is considered equivalent to approximately 10 years of natural aging at 37 °C in a clinical setting. The selected aging duration was determined based on previous studies simulating long-term environmental stresses on zirconia restorations [10, 12].

Following the LTD procedure, specimens were immersed in different pH solutions at 37 °C for 14 days to evaluate their chemical stability:

- pH 3 Group (Acidic Environment): Specimens were stored in a pH 3 acidic buffer solution to simulate exposure to acidic beverages and gastric acid. The solution was prepared by dissolving 21.0 g citric acid in 200 mL of 1 M sodium hydroxide solution, then adjusting the volume to 1000 mL.
- pH 9 Group (Basic Environment): Specimens were stored in a pH 9 basic buffer solution to simulate the alkaline effects of toothpaste and mouthwashes. The solution was prepared using ammonium hydroxide (NH₄OH) and ammonium chloride (NH₄Cl).

pH levels were monitored and adjusted daily to ensure solution stability during the 14-day immersion.

### Mechanical and surface characterization

The mechanical and surface properties of the specimens were evaluated following ISO 6872 and ISO 13,356 standards [21, 22]. These analyses aimed to assess biomechanical strength, surface roughness, and crystalline phase transformations, enabling the comparison of the effects of different surface treatments and aging conditions.

### Biaxial flexural strength (BFS) testing

The biaxial flexural strength (BFS) of the specimens was measured according to ISO 6872 using the piston-on-three-ball test. The tests were conducted on a universal testing machine (Instron 5965, Instron Corp., USA) with a crosshead speed of 1 mm/min, and the fracture load (F) was recorded. The BFS was calculated using the following equation:

$$\sigma\_f=XF/h^\wedge2$$

- σ_f = Biaxial flexural strength (MPa)
- F = Fracture load (N)
- h = Specimen thickness (mm)
- X = Geometric factor depending on support and loading diameters

This test was conducted to determine the fracture resistance of the specimens and evaluate their mechanical performance under clinically relevant loading conditions.

### Weibull analysis

The obtained biaxial flexural strength data were analyzed using the Weibull distribution to assess the reliability and homogeneity of the specimens. The characteristic strength (σ₀) and Weibull modulus (m) were calculated to determine the failure probability and the variability in strength distribution. The Weibull cumulative failure probability was defined by the following equation:

$$P\_f=1-e^\wedge\left(-\left(\sigma/\sigma_0\right)^\wedge m\right)$$

- P_f = Probability of failure
- σ = Experimentally measured flexural strength (MPa)
- σ₀ = Characteristic flexural strength (MPa)
- m = Weibull modulus

Weibull analysis was performed to evaluate the effects of different surface treatments and aging conditions on the mechanical reliability of the zirconia specimens. A higher Weibull modulus (m) indicates greater homogeneity and reliability, whereas a lower m value suggests greater variability in mechanical strength.

### Phase analysis by X-Ray diffraction (XRD)

X-ray diffraction (XRD) analysis was performed using a diffractometer (Empyrean, Malvern Panalytical, Netherlands) with Cu Kα radiation (λ = 1.5406 Å) at 40 kV and 40 mA. Data were collected in the 20°–90° 2θ range with a step size of 0.02° and a scanning speed of 1°/min. Rietveld refinement was performed using a pseudo-Voigt profile, and the phase model included tetragonal, cubic, and monoclinic zirconia phases. Polishing-induced rhombohedral phase was not detected in our specimens.

X-ray diffraction (XRD) analysis was performed using Cu Kα radiation. The monoclinic phase fraction was calculated according to the Garvie and Nicholson method [23], corrected by Toraya et al. [24], using the following formulas:

$$Xm=\left[Im\left(-111\right)+Im\left(111\right)\right]/\left[Im\left(-111\right)+Im\left(111\right)+It\left(101\right)\right]$$

$$Vm=\left[1.311\times Xm\right]/\left[1+0.311\times Xm\right]$$

It: Intensity of the tetragonal phase peak; Im: Intensity of the monoclinic phase peaks (− 111 and 111).

This analysis was conducted to assess the phase transformations in the crystalline structure after low-temperature degradation (LTD) and to investigate the effect of different pH environments on phase stability.

### Surface morphology analysis by scanning electron microscopy (SEM)

The surface morphologies of the specimens were examined in detail using a scanning electron microscope (SEM, Quanta 250 FEG, Thermo Fisher Scientific, USA). This analysis aimed to evaluate the effects of different surface treatments and aging protocols on the surface microstructure. To enhance electrical conductivity and prevent surface charging, the specimens were coated with a 10 nm thick gold-palladium (Au-Pd) layer. The coating process was performed under low vacuum using a sputter coater (Leica EM ACE600, Leica Microsystems, Germany). Following the coating, each specimen was affixed to a metal SEM stub using carbon tape and analyzed under a vacuum environment. SEM imaging was conducted at a 20 kV accelerating voltage, a 10 mm working distance, and various magnifications. In particular, 1000×, and 2500× magnifications were utilized to examine surface morphology in detail.

### Surface roughness measurements

Surface roughness measurements were performed using a white light interferometric profilometer (Talysurf CCI HD, Taylor Hobson, UK) to quantitatively assess the impact of surface treatments and aging conditions on surface morphology. Average roughness (Ra) and root mean square roughness (Rq) values were analyzed to evaluate the effects of grinding, polishing, and glazing on surface smoothness and homogeneity. Measurements were conducted in non-contact mode to prevent mechanical damage, with a 2.5 mm × 2.5 mm scanning area, 0.01 μm vertical resolution, 0.5 μm lateral resolution, Gaussian filtering (λc = 0.08 mm), and a scanning speed of 1.0 mm/s. Each specimen was measured at five randomly selected points, and automatic focusing and precision height adjustment were used to ensure data accuracy.

### Statistical analysis

The biaxial flexural strength, surface roughness (Ra, Rq), and XRD phase transformation data were statistically analyzed to evaluate the effects of different surface treatments and aging conditions on the specimens. Shapiro-Wilk test was used to assess the normality of the data, while Levene’s test evaluated variance homogeneity. For normally distributed data, one-way ANOVA was applied, followed by Tukey HSD post-hoc test when significant differences were detected. The reliability and durability of biaxial flexural strength were assessed using Weibull analysis, where the characteristic strength (σ₀) and Weibull modulus (m) were calculated. Differences in monoclinic phase fractions obtained from XRD analyses were examined using the Kruskal-Wallis test, while correlations between biaxial flexural strength and phase transformation were analyzed using Pearson or Spearman correlation tests.

All statistical analyses were conducted using SPSS (IBM Corp., USA) and OriginPro (OriginLab Corp., USA), while Weibull and phase transformation calculations were performed using MATLAB (MathWorks, USA) or Python (SciPy library). The significance level was set at *p* < 0.05.

## Results

**Fig. 1:**
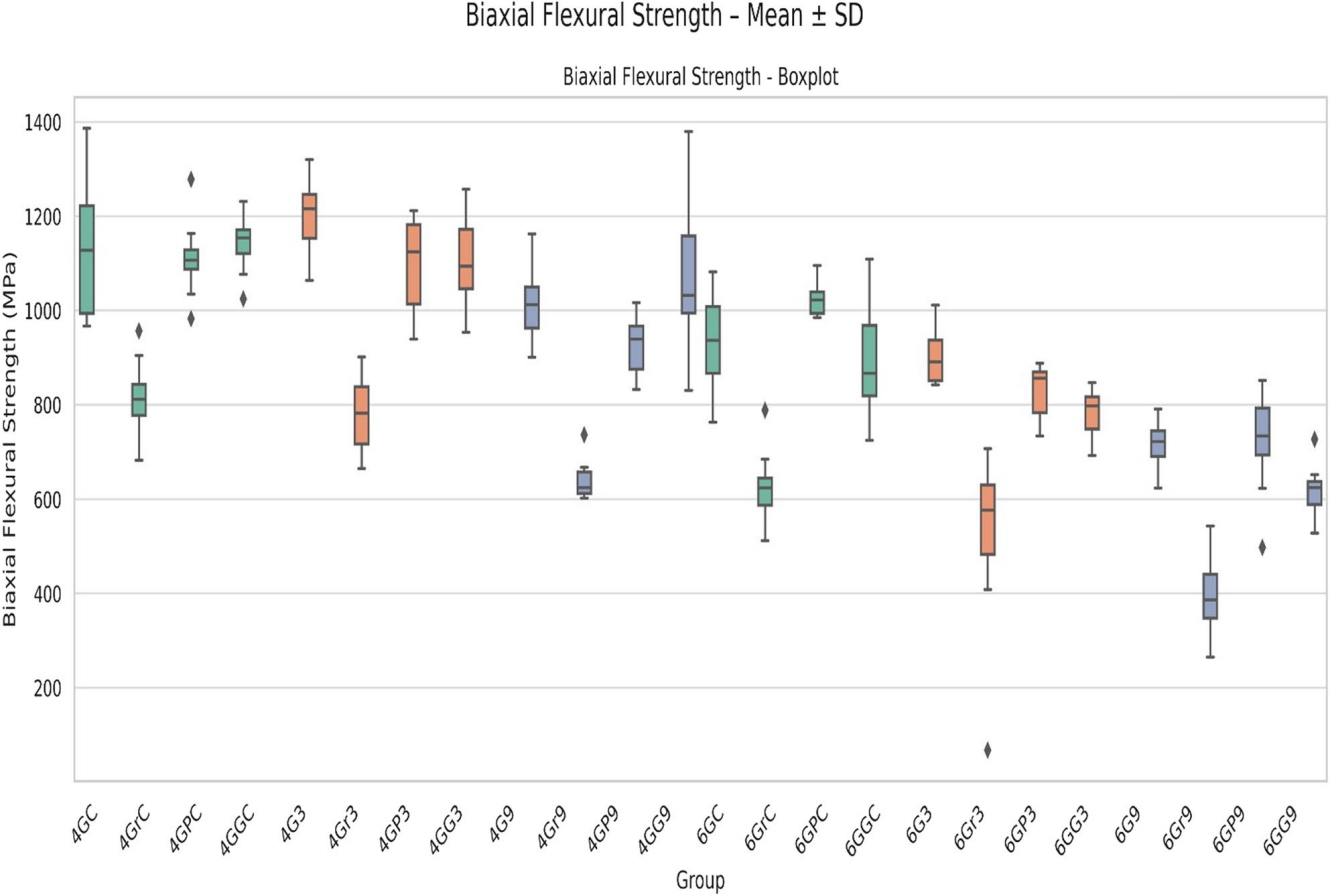
Biaxial flexural strength distribution of all experimental groups. The box plots represent median, interquartile range, and minimum–maximum values for each group. Notable differences are observed among surface treatments and aging conditions for both 4Y-TZP and 6Y-TZP zirconia

The biaxial flexural strength values exhibited significant differences across all groups, depending on the surface treatments and LTD application (*p* < 0.05) (Table 3). The grinding + polishing group showed flexural strength than the control group, partially compensating for the loss due to grinding, while the grinding-only group had the lowest mechanical strength. The grinding + glazing group exhibited intermediate strength values, slightly above the control group but lower than polishing. LTD led to a reduction in strength across all groups most prominently in the pH 9 subgroups. Significant differences in biaxial flexural strength were observed between 4Y-TZP and 6Y-TZP specimens (*p* < 0.05), with the highest values recorded in the 4G3 and 6G3 groups, while the lowest values were found in the 4Gr9 and 6Gr9 groups. The mechanical strength decreased considerably after grinding, whereas polishing (Grinded + Polished) partially restored the strength (Fig. 1).

**Table 3:** Biaxial flexural strength, surface roughness, and XRD analysis results for each experimental group

	Biaxial flexural strength				Surface Roughness		XRD Analysis(%)	
	Descriptive Analysis	Weibull Analysis						
Group	Mean ± SD (MPa)		Weibull Modulus (m)	Weibull Scale (σ₀)	Mean ± SD (Ra)	Mean ± SD (Rq)	t-phase	c-phase
4GC	1171.4 ± 113.3		1220.25	12.5	0.71 ± 0.12	1.07 ± 0.63	78.2	21.8
4GrC	839.3 ± 72.9		871.12	14.0	1.98 ± 0.44	2.46 ± 0.70	75.4	24.6
4GPC	1111.6 ± 118.3		1163.07	10.9	0.92 ± 0.36	1.35 ± 0.59	79.0	21.0
4GGC	1137.5 ± 98.9		1181.24	13.2	1.17 ± 0.22	1.65 ± 0.72	77.9	22.1
4G3	1217.5 ± 81.5		1252.71	20.0	0.99 ± 0.37	1.14 ± 0.36	79.5	20.5
4Gr3	760.5 ± 79.5		794.46	11.4	2.29 ± 0.42	2.66 ± 0.72	73.5	26.5
4GP3	1095.5 ± 89.1		1133.89	15.9	1.48 ± 0.24	1.78 ± 0.96	78.7	21.3
4GG3	1119.9 ± 98.7		1164.18	12.3	1.47 ± 0.29	1.56 ± 0.49	80.1	19.9
4G9	1014.3 ± 91.7		1056.3	11.3	1.61 ± 0.20	1.64 ± 0.23	76.3	23.7
4Gr9	631.2 ± 65.7		660.46	10.1	2.57 ± 0.31	2.65 ± 0.57	72.1	27.9
4GP9	938.0 ± 67.1		966.11	19.9	1.60 ± 0.43	1.95 ± 1.03	74.8	25.2
4GG9	1076.2 ± 136.3		1134.67	9.0	1.80 ± 0.43	2.14 ± 0.60	75.5	24.5
6GC	1171.4 ± 113.3		1220.25	12.5	0.71 ± 0.12	1.07 ± 0.63	65.8	34.2
6GrC	839.3 ± 72.9		871.12	14.0	1.98 ± 0.44	2.46 ± 0.70	62.2	37.8
6GPC	1111.6 ± 118.3		1163.07	10.9	0.92 ± 0.36	1.35 ± 0.59	64.3	35.7
6GGC	1137.5 ± 98.9		1181.24	13.2	1.17 ± 0.22	1.65 ± 0.72	65.2	34.8
6G3	1217.5 ± 81.5		1252.71	20.0	0.99 ± 0.37	1.14 ± 0.36	67.2	32.8
6Gr3	760.5 ± 79.5		794.46	11.4	2.29 ± 0.42	2.66 ± 0.72	61.4	38.6
6GP3	1095.5 ± 89.1		1133.89	15.9	1.48 ± 0.24	1.78 ± 0.96	66.7	33.3
6GG3	1119.9 ± 98.7		1164.18	12.3	1.47 ± 0.29	1.56 ± 0.49	66.9	33.1
6G9	1014.3 ± 91.7		1056.3	11.3	1.61 ± 0.20	1.64 ± 0.23	64.5	35.5
6Gr9	631.2 ± 65.7		660.46	10.1	2.57 ± 0.31	2.65 ± 0.57	60.8	39.2
6GP9	938.0 ± 67.1		966.11	19.9	1.60 ± 0.43	1.95 ± 1.03	62.9	37.1
6GG9	1076.2 ± 136.3		1134.67	9.0	1.80 ± 0.43	2.14 ± 0.60	63.1	36.9

*Abbreviations*: Arithmetic mean ± standard deviation, *Ra* Arithmetic average of absolute values of surface height deviations, *Rq* Root mean square of surface height deviations, *t-phase* Tetragonal phase (%), *c-phase* Cubic phase (%), *Weibull shape (m)* Distribution shape parameter indicating variability, *Weibull scale (σ₀)* Characteristic strength indicating reliability

Outliers observed in Fig. 1 were retained in the analysis, as they originated from valid specimens rather than preparation errors, and statistical robustness was confirmed by normality and homogeneity tests.

**Fig. 2:**
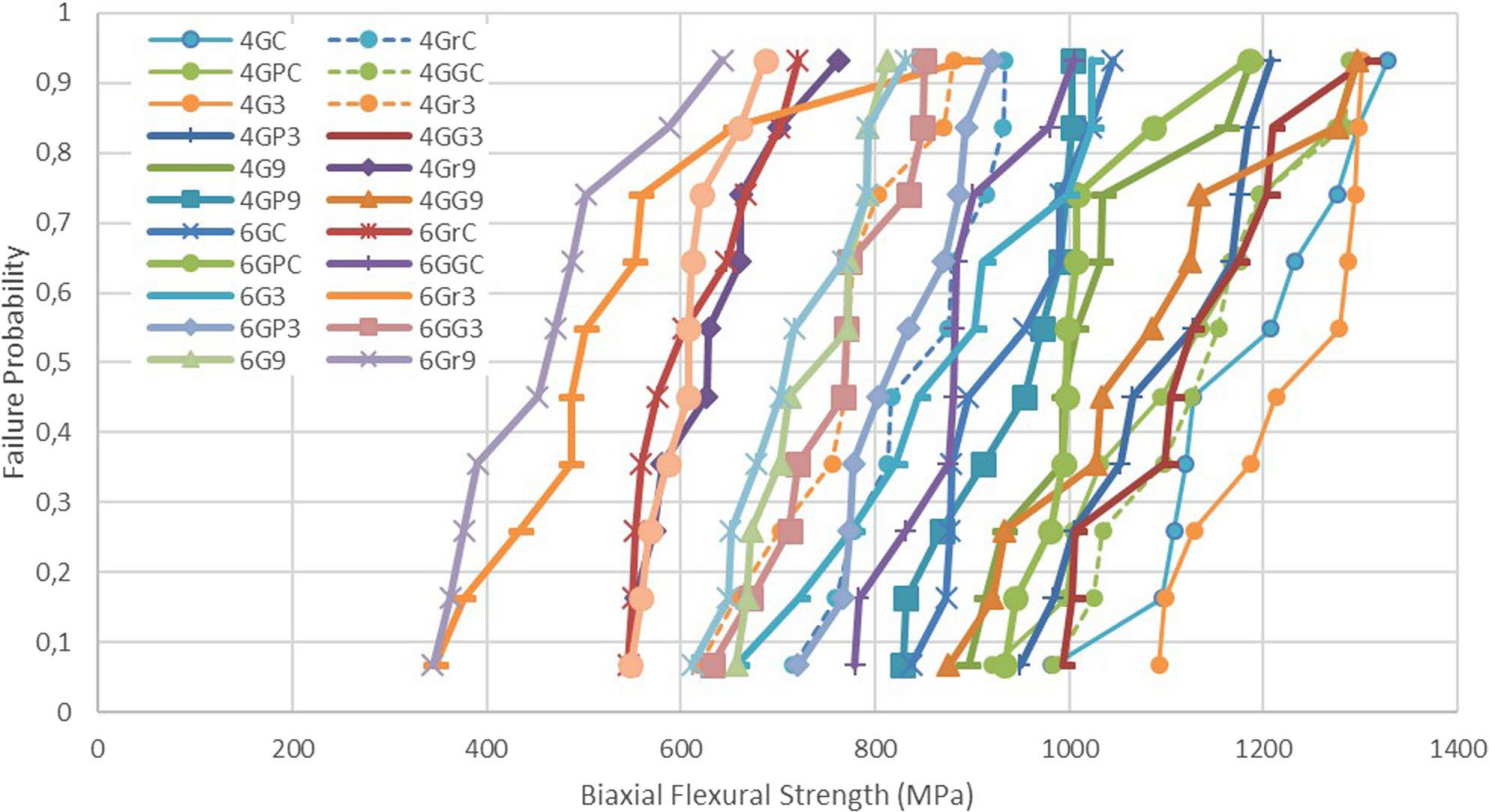
Weibull distribution plot of biaxial flexural strength for all experimental groups. The plot shows the relationship between log-transformed strength values and their cumulative failure probabilities (log[-log(1–CDF)]). Each dot represents an individual specimen, and groups are color-coded. The linearity of data points reflects the reliability and variability of sddtrength within each group

Weibull analysis revealed variations in characteristic strength (σ₀) and reliability (m) values depending on surface treatments. The Weibull modulus and scale values for all groups are presented in Table 3. In general, higher m values indicated greater homogeneity and reliability (e.g., polished and glazed groups), while lower m values (e.g., ground groups, particularly after LTD) reflected increased variability in mechanical performance (Fig. 2; Table 3).

**Fig. 3:**
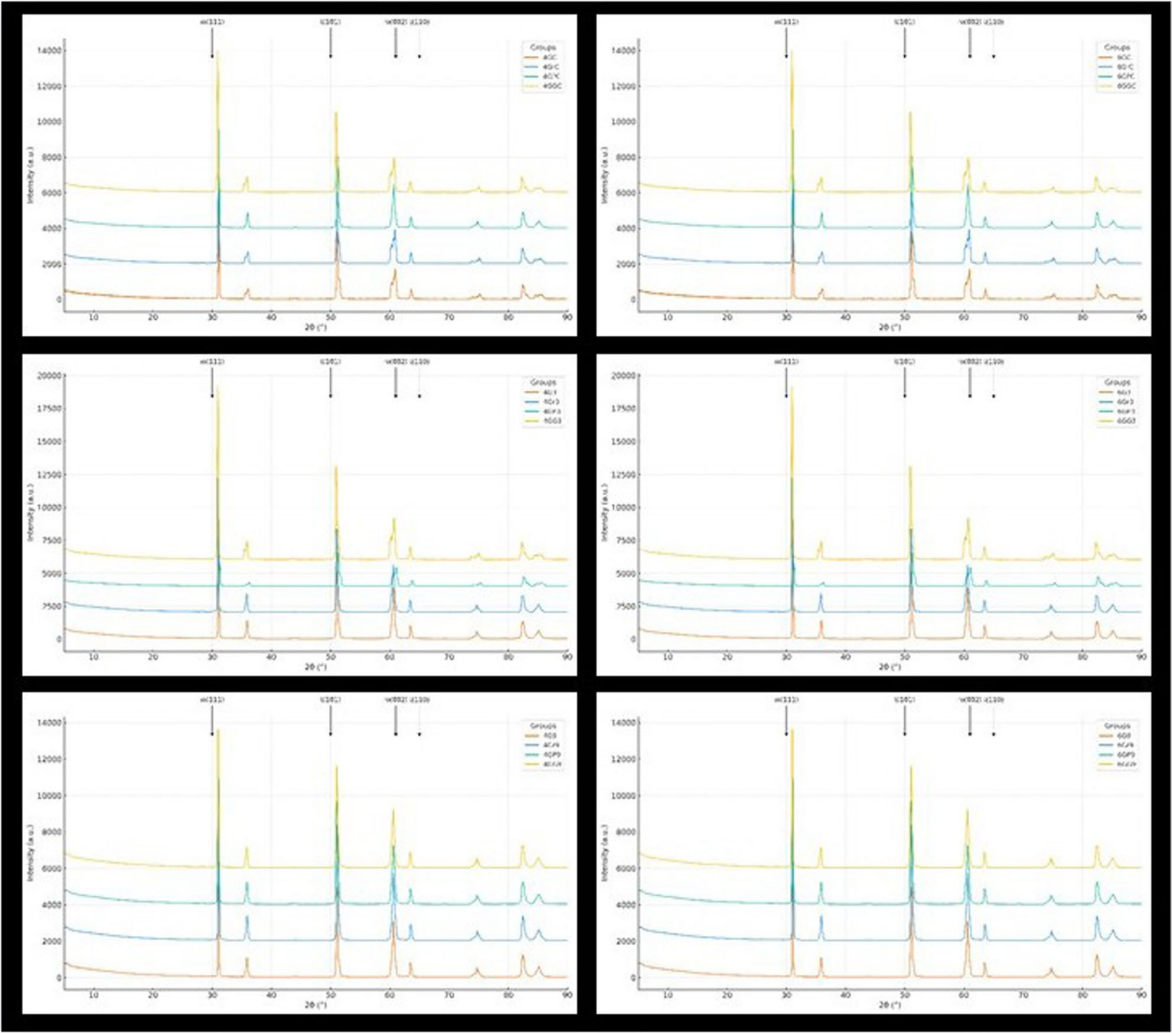
Representative XRD patterns of 4Y-TZP and 6Y-TZP specimens after different surface treatments (glazed, grinded, grinded + polished, grinded + glazed) and aging conditions (distilled water storage, LTD + pH 3, LTD + pH 9). Insets highlight the 26–36° 2θ region, emphasizing t(101) and m(111) reflections associated with phase transformation

XRD analyses demonstrated an increase in monoclinic phase content after LTD (Fig. 3). The monoclinic phase fraction, initially 3% in the control group, increased to 18% post-LTD. The grinding + polishing specimens exhibited the lowest monoclinic phase transformation (12%), whereas the grinding-only group showed the highest increase (25%). Across all groups, LTD led to a significant increase in monoclinic phase content, particularly in ground specimens (*p* < 0.05), though polishing mitigated this effect. In glazed specimens, monoclinic phase content remained lower than in polished samples. Additionally, 4Y-TZP samples exhibited greater monoclinic transformation than 6Y-TZP, likely due to higher cubic phase content in 6Y-TZP. A negative correlation was observed between monoclinic phase content and biaxial flexural strength, with mechanically weaker groups displaying higher monoclinic phase content. Notably, in the 4Gr9 and 6Gr9 groups, the significant reduction in flexural strength correlated with extensive monoclinic transformation (*p* < 0.05).

**Fig. 4:**
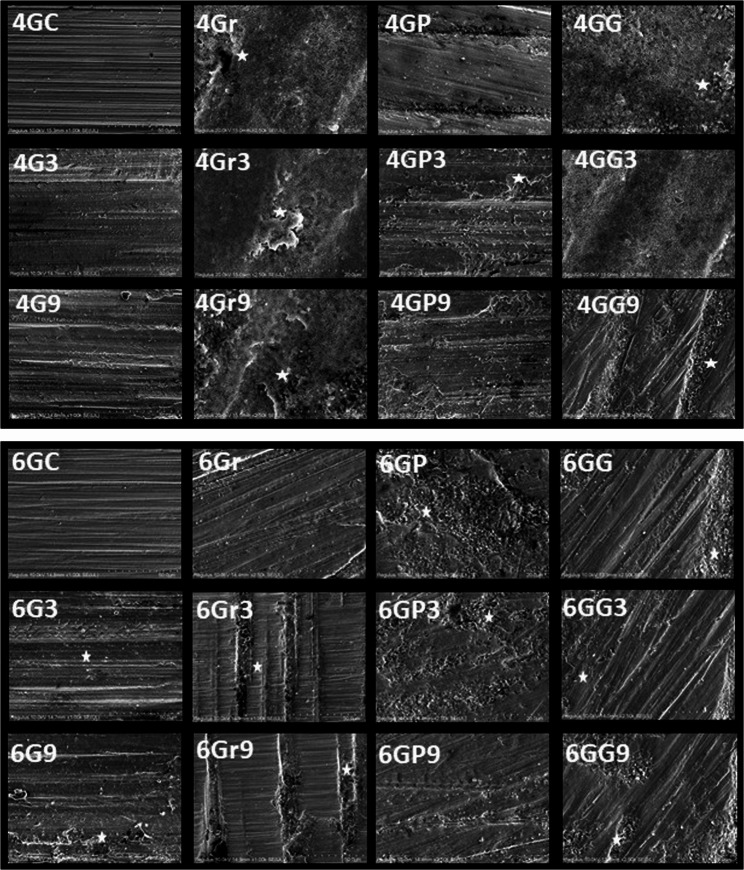
Representative SEM images (20 kV, WD = 10 mm, SE detector, 1000×/2500×) showing surface morphologies of 4Y-TZP and 6Y-TZP specimens after different surface treatments. The areas where the effects of aging are visible have been marked with asterisks

Surface roughness (Ra and Rq) analysis revealed statistically significant differences among the groups depending on surface treatments and environmental conditions (*p* < 0.05). The lowest surface roughness values were observed in the glazed control (GC) groups for both 4Y-TZP (Ra: 0.71 ± 0.12 μm; Rq: 1.07 ± 0.63 μm) and 6Y-TZP (Ra: 0.71 ± 0.12 μm; Rq: 1.07 ± 0.63 μm) specimens. In contrast, the grinding-only groups exhibited the highest roughness, particularly in 4Gr9 (Ra: 2.57 ± 0.31 μm; Rq: 2.65 ± 0.57 μm) and 6Gr9 (Ra: 2.57 ± 0.31 μm; Rq: 2.65 ± 0.57 μm). Polishing after grinding significantly reduced surface roughness. In the 4GP3 and 6GP3 groups, Ra values were measured at 1.48 ± 0.24 μm and 1.48 ± 0.24 μm, respectively. Glazing after grinding was also effective but slightly less so than polishing. Furthermore, acidic conditions consistently increased surface roughness in all groups. For instance, Ra in the 4GG3 group was 1.47 ± 0.29 μm, while in 4GG9 it increased to 1.80 ± 0.43 μm. (Table 3) The SEM micrographs reveal distinct surface morphologies depending on the treatment applied. Control groups (e.g., 4GC, 6GC) exhibit relatively smooth surfaces with minimal topographical features, corresponding to the lowest Ra and Rq values. In contrast, grit-blasted specimens (e.g., 4Gr9, 6Gr9) display highly irregular and rough surfaces with prominent scratches and grooves, consistent with their high roughness measurements. Polished and ground groups (GP, GG) show intermediate roughness, characterized by directional patterns and surface debris. Overall, the surface modifications significantly altered the topography, which may influence mechanical performance and phase composition, as corroborated by the surface roughness and XRD data (Fig. 4).

## Discussion

This work aimed to comprehensively assess the mechanical strength and surface modifications of several Y-TZP zirconia materials after low-temperature deterioration (LTD). The study hypothesis posits that different surface treatments will significantly affect mechanical performance, with post-grinding polishing expected to improve mechanical strength and reduce surface roughness. Nevertheless, the findings partially contradict this hypothesis. While post-grinding polishing significantly decreased surface roughness and improved mechanical strength to a degree, it did not entirely offset the mechanical losses caused by the grinding process. Moreover, all groups demonstrated an increase in monoclinic phase change following LTD, which adversely impacted mechanical strength. These findings underscore that surface changes alone are unable to improve mechanical performance and highlight the significant impact of environmental variables, such as LTD, on zirconia stability.

The post-grinding polishing procedure markedly decreased surface roughness and improved flexural strength in this study. Specimens that underwent just grinding shown a significant reduction in mechanical strength, as surface imperfections initiated crack development and heightened fracture susceptibility. This result aligns with recent research suggesting that microstructural alterations caused by grinding might adversely affect the material’s resistance mechanisms [3, 4]. Moreover, glazing partly stabilized the surface and restricted fracture propagation; nevertheless, it did not completely avert strength degradation following LTD. The results of the glazing-treated specimens indicate that while the glass-like structural layer offers some protection, it is not entirely resistant to mechanical loads and high temperatures [25–27] Phatphutthitham et al. [28] reported that adherence to standardized polishing protocols significantly enhances surface quality in high-translucent zirconia, which is in concordance with the present study, wherein post-grinding polishing achieved the lowest surface roughness and highest biaxial flexural strength. Nonetheless, polishing could not fully compensate for the mechanical deficits induced by prior grinding, underscoring its limitations as a standalone corrective intervention. Similarly, findings by Alves et al. [25] have shown that glazing may reduce surface wear but remains insufficient in preventing subsurface degradation under hydrothermal conditions. While our study did not include combined treatments (e.g., polishing + glazing), recent literature suggests these may offer enhanced protection. Compared to these studies, our work contributes additional insight by incorporating long-term pH exposure, revealing that acidic environments significantly accelerate phase transformation and mechanical degradation—an aspect often overlooked in earlier research. Current research has investigated the influence of pH on monoclinic phase change and mechanical strength via short-term assessments, neglecting the implications of extended chemical exposure, offering a full understanding of zirconia restorations’ behavior under environmental influences. Our results corroborate earlier research, indicating that polishing subsequent to grinding diminishes microcracks and stress buildup, thereby enhancing mechanical strength [29, 30].

In addition to the optimization of surface treatments, ensuring chemical stability is a critical factor in improving the clinical performance of zirconia restorations [8, 9, 14]. Among the various surface modifications evaluated following low-temperature degradation (LTD), post-grinding polishing proved to be the most effective, resulting in the highest flexural strength and the lowest surface roughness. The findings indicate that polishing is the most efficacious surface treatment for preserving the long-term durability of zirconia restorations and should be emphasized in clinical practice. In this study, the monoclinic phase transformation rate attained 22% in a pH 3 environment, whereas it was constrained to 15% in a pH 9 condition. Similar to our findings, Alnasser et al. [14] reported that acidic pH cycling significantly accelerates surface degradation and reduces flexural strength in monolithic zirconia, further supporting the negative impact of low-pH environments observed in our study following LTD. Regarding flexural strength, specimens maintained at pH 3 exhibited a drop of up to 30%, while those in a pH 9 environment demonstrated an 18% loss. It was indicated that acidic circumstances expedite the tetragonal-to-monoclinic phase shift, hence enhancing microcrack development and diminishing flexural strength, and was shown that polishing alleviates this impact by reducing residual stress and surface roughness. Likewise, Sulaiman et al. [31] discovered that monolithic zirconia restorations exhibit increased vulnerability to surface deterioration caused by acidic conditions. Fadavi et al. [12] indicated that neutral surroundings have a protective benefit relative to acidic circumstances, and our research corroborates these findings by showing that LTD-related degradation is reduced at pH 9 compared to pH 3. This effect can be ascribed to the heightened presence of hydroxyl groups on the zirconia surface in alkaline conditions, which diminishes the infiltration of water molecules into the material.

SEM and profilometry investigations demonstrate that acidic conditions result in heightened surface erosion and the development of cracks. Post-grinding polishing of specimens resulted in surface uniformity and significantly mitigated fracture development. The Ra value for the polished group was recorded at 0.6 ± 0.1 μm, but it was markedly elevated in the grinding-only group at 1.8 ± 0.2 μm. In the glazing-treated group, surface roughness was reduced relative to the control group, however it was less successful than polishing (Ra = 1.2 ± 0.1 μm). Surface roughness study indicated a substantial increase post-grinding, while polishing significantly decreased roughness (*p* < 0.05). Corcodel et al. [1] indicated that grinding generates microcracks on the zirconia surface, undermining mechanical stability, whereas polishing partially rectifies these imperfections and enhances strength. Weibull analysis results support these conclusions; groups who did not polish showed more variability in strength distribution and a lower Weibull modulus (m). Conversely, a more uniform strength distribution is indicated by a polished specimen’s increased Weibull modulus [2]. Nevertheless, polishing alone was unable to completely restore surface integrity. The retention of microcracks post-grinding led to decreased mechanical strength in certain groups. Hatanaka et al. [27] observed that microcracks may persist on the zirconia surface post-polishing, possibly spreading under clinical stresses and undermining repair durability. Lee et al. [32] demonstrated that while both glazing and polishing influence the surface characteristics of translucent zirconia, polishing was more effective in limiting phase transformation. Their findings support our observation that post-grinding polishing not only improves surface smoothness but also helps reduce monoclinic phase formation, thereby enhancing the material’s structural stability. Caglar et al. [33] established that grinding induces phase change in zirconia, resulting in an increase in monoclinic phase content. In our investigation, LTD further augmented the fraction of the monoclinic phase across all groups, with this impact being more obvious in specimens subjected to grinding treatment. The observed reduction in monoclinic content after polishing is consistent with the mitigation of grinding-induced defects and residual stresses by polishing [34, 35].

The high-cubic-content 6Y-TZP demonstrated enhanced resistance to phase transition during low-temperature degradation. The augmentation of monoclinic phase composition during LTD markedly diminished mechanical strength, although polishing slightly alleviated this change. The noted decrease in monoclinic phase change following polishing may be ascribed to the mitigation of surface tensions. Additionally, 4Y-TZP exhibited a greater monoclinic increase than 6Y-TZP, consistent with the higher cubic fraction and LTD resistance reported for higher-yttria zirconias [34–37]. Consequently, the use of 6Y-TZP in high-load-bearing regions need careful consideration, and surface treatments must be chosen to enhance mechanical performance. Reducing surface roughness in monolithic zirconia restorations may improve their long-term efficacy [3–5]. Moreover, acidic environments were seen to expedite mechanical deterioration in zirconia restorations, underscoring the necessity to restrict extended contact to these circumstances. The decreased deterioration seen in the pH 9 environment indicates that somewhat alkaline circumstances may aid in preserving restorative stability [10, 12]. pH markedly influences degradation: acidic media can promote yttria loss and accelerate the tetragonal-to-monoclinic transformation, whereas alkaline exposure alters surface hydroxylation and may modulate aging kinetics [38]. One limitation of this study is the exclusion of dynamic intraoral conditions such as masticatory forces, thermal cycling, and the presence of oral biofilms, all of which can significantly influence the long-term behavior of zirconia restorations. While the in vitro protocols, including LTD and pH exposure, simulate certain aging processes, they do not fully replicate the complex mechanical and chemical environment of the oral cavity. Studies have shown that thermal fluctuations and repetitive loading can exacerbate microcrack propagation and phase transformation in zirconia, while biofilm activity may alter surface chemistry and accelerate degradation [8–10, 12]. The integration of these characteristics into future research may offer a more clinically pertinent comprehension of the longevity of restorations in practical settings.

## Conclusion

- Polishing is critical for ensuring the longevity of zirconia restorations, as it improves mechanical strength and reduces surface roughness after grinding.
- When grinding is unavoidable, polishing must be performed; otherwise, the risk of early mechanical failure in zirconia restorations increases.
- Monoclinic phase transformation after LTD varies depending on surface treatments, with 6Y-TZP showing lower phase transformation and greater resistance to aging.
- To enhance the clinical success of zirconia restorations in high-load posterior regions, surface treatments should be carefully selected.

## Supplementary Information

Supplementary Material 1.

## Data Availability

The datasets used and/or analysed during the current study are available from the corresponding author on reasonable request.

## Abbreviations

LTD: low-temperature degradation
Y-TZP: yttria-stabilized tetragonal zirconia polycrystals
Y₂O₃: yttria
NH₄OH: ammonium hydroxide
NH₄Cl: ammonium chloride
BFS: biaxial flexural strength
XRD: X-ray diffraction
SEM: Scanning Electron Microscopy
GC: Glazed-Control
GrC: Grinded-Control
GPC: Grinded + Polished- Control
GGC: Grinded + Glazed- Control
G3: Glazed- pH3
Gr3: Grinded- pH3
GP3: Grinded + Polished- pH3
GG3: Grinded + Glazed- pH3
G9: Glazed- pH9
Gr9: Grinded- pH9
GP9: Grinded + Polished- pH9
GG9: Grinded + Glazed- pH9
