# Supplementary material for: Effects of surface treatments, LTD aging, and pH on the mechanical behavior of high-translucent zirconia
Source: BMC Oral Health. 2025 Dec 10;26:51. doi: 10.1186/s12903-025-07473-2 (PMC12790711; doi:10.1186/s12903-025-07473-2)
Supplement: Supplementary file 1 — Supplementary Material 1. [file 12903_2025_7473_MOESM1_ESM.docx]

**Figure Legends**

**Figure 1.** Biaxial flexural strength distribution of all experimental groups. The box plots represent median, interquartile range, and minimum–maximum values for each group. Notable differences are observed among surface treatments and aging conditions for both 4Y-TZP and 6Y-TZP zirconia.

**Figure 2.** Weibull distribution plot of biaxial flexural strength for all experimental groups. The plot shows the relationship between log-transformed strength values and their cumulative failure probabilities (log[-log(1–CDF)]). Each dot represents an individual specimen, and groups are color-coded. The linearity of data points reflects the reliability and variability of sddtrength within each group**.**

**Figure 3**. Representative XRD patterns of 4Y-TZP and 6Y-TZP specimens after different surface treatments (glazed, grinded, grinded + polished, grinded + glazed) and aging conditions (distilled water storage, LTD + pH 3, LTD + pH 9). Insets highlight the 26–36° 2θ region, emphasizing *t*(101) and *m*(111) reflections associated with phase transformation.

**Figure 4.** Representative SEM images (20 kV, WD = 10 mm, SE detector, 1000×/2500×) showing surface morphologies of 4Y-TZP and 6Y-TZP specimens after different surface treatments. The areas where the effects of aging are visible have been marked with asterisks.
